# Broad and Effective Protection against Staphylococcus aureus Is Elicited by a Multivalent Vaccine Formulated with Novel Antigens

**DOI:** 10.1128/mSphere.00362-19

**Published:** 2019-09-04

**Authors:** Jian Deng, Xiaolei Wang, Bao-Zhong Zhang, Peng Gao, Qiubin Lin, Richard Yi-Tsun Kao, Kenth Gustafsson, Kwok-Yung Yuen, Jian-Dong Huang

**Affiliations:** a School of Biomedical Sciences, University of Hong Kong, Hong Kong, China; b Shenzhen Institute of Advanced Technologies, Shenzhen, China; c Department of Microbiology, University of Hong Kong, Hong Kong, China; d State Key Laboratory of Emerging Infectious Diseases, University of Hong Kong, Hong Kong, China; e UCL Institute of Child Health, Molecular Immunology Unit, London, United Kingdom; f HKU-Shenzhen Institute of Research and Innovation, Shenzhen, China; U.S. Food and Drug Administration

**Keywords:** γδ T cell, MRSA, multivalent vaccine

## Abstract

The demand for a prophylactic vaccine against methicillin-resistant Staphylococcus aureus (MRSA) has motivated numerous dedicated research groups to design and develop such a vaccine. In this study, we have developed a multivalent vaccine, Sta-V5, composed of five conserved antigens involved in three important virulence mechanisms. This prototype vaccine conferred up to 100% protection against multiple epidemiologically relevant S. aureus isolates in five different murine disease models. The vaccine not only elicits functional antibodies that mediate opsonophagocytic killing of S. aureus but also mounts robust antigen-specific T-cell responses. In addition, our data implied that γδ T cells contribute to the protection induced by Sta-V5 in a murine skin infection model.

**IMPORTANCE** Staphylococcus aureus infections, especially MRSA infections, are becoming a major global health issue and are resulting in mortality rates that are increasing every year. However, an effective vaccine is lacking due to the complexity of the infection process of S. aureus. In this study, we found that the addition of two novel protein components to three well-studied vaccine candidates significantly improved the efficacy of the combined vaccine. Furthermore, the five-component vaccine not only elicits a robust antibody response but also induces cytokine secretion by T cells, making it a promising vaccine candidate to fill the void.

## Abstract (precis)

Staphylococcus aureus infections, especially MRSA infections, are becoming a major global health issue and are resulting in mortality rates that are increasing every year. However, an effective vaccine is lacking due to the complexity of the infection process of S. aureus. In this study, we found that the addition of two novel protein components to three well-studied vaccine candidates significantly improved the efficacy of the combined vaccine. Furthermore, the five-component vaccine not only elicits a robust antibody response but also induces cytokine secretion by T cells, making it a promising vaccine candidate to fill the void.

## INTRODUCTION

Staphylococcus aureus is a facultative anaerobic Gram-positive bacterium, frequently found as part of the normal flora on the skin and in the nasal passages (1, 2). S. aureus can cause a range of illnesses from minor skin infections (e.g., pimples, cellulitis folliculitis, carbuncles, scalded skin syndrome, and abscesses) to life-threatening diseases (e.g., meningitis, pneumonia, toxic shock syndrome, bacteremia, and sepsis). The emergence of antibiotics-resistant S. aureus strains (for example, methicillin-resistant S. aureus [MRSA]) has made it one of the most dangerous pathogens infecting humans. As an alternative strategy, preventative vaccination is one of the most promising approaches to combat MRSA without any concerns regarding antibiotic resistance (3). Previous studies have identified a myriad of virulence factors from S. aureus. Many of these virulence factors, along with surface proteins, have been evaluated as potential targets for vaccines (4). Alpha-toxin (5), clumping factor A (ClfA) (6), fibronectin binding protein (FnBPA or FnBPB), Panton-Valentine leukocidin (PVL) (7), and protein A (8) and iron-regulated proteins such as IsdB (9) have been investigated as vaccine targets. The leading effort applied to studies conducted with these types of vaccine has been associated with StaphVAX, a bivalent polysaccharide-protein conjugate vaccine (10). However, the efficacies of vaccines based on the antigens mentioned above have yet to be improved. So far, all active and passive immunotherapies developed against MRSA have failed to show efficacy in clinical trials, presumably because these vaccines provide protection against only a fraction of the complex immune evasion strategies taken by S. aureus (11). However, recent efforts, including our work, have produced very promising results (12, 13). Because S. aureus adopts various complex strategies to evade or interfere with the immune response of the host, the key to developing an effective vaccine against this pathogen is the inclusion of antigens that target multiple virulence mechanisms involved in the establishment and maintenance of infection (14). In line with this strategy, we launched a vaccine discovery project in search of conserved antigens. These antigens are combined to formulate a multivalent vaccine according to the following criteria. (i) Each antigen is conserved and widely expressed among variety of clinical isolates. (ii) Each antigen plays important roles in pathogenesis of S. aureus. As a result, we have formulated a multivalent vaccine composed of five antigens—recombinant AdsA (rAdsA), EsxA/B, and PmtA/C. On the other hand, it has been argued on the basis of past failures of human clinical trials (15, 16) that mouse models cannot accurately identify the protective antigens of S. aureus and therefore are not predictive of human responses. However, mouse models remain the most important surrogates as the first step for the study of staphylococcal infections in humans and represent the best cost-to-value ratio (16). In this study, five mouse models which reflect the progression of several diseases, including skin infection, blood infection, kidney infection, peritonitis, and pneumonia, were used to comprehensively evaluate the efficacy of this multivalent vaccine.

## RESULTS

### Selection of antigens.

Among the antigens known as pivotal virulence factors involved in pathogenesis of S. aureus, we selected five proteins to formulate our candidate combination vaccine. Two of them, EsxA and EsxB, are secreted by a specialized secretion system termed the ESAT-6-like system. Mutants that fail to secrete EsxA and EsxB do not cause S. aureus*-*induced murine abscesses (17). Our recent study showed that EsxA and EsxB, can potentially be used as vaccine antigens against S. aureus (13). This observation was further supported by another study published shortly afterward (12). AdsA, a cell wall-anchored protein that was recently identified as an essential immune evasion factor (18), is also included in our formulation. A previous study performed in our laboratory demonstrated that mice vaccinated with recombinant AdsA (rAdsA) induced high titers of anti-AdsA antibodies and provided consistent protection in three mouse infection models under conditions of challenge with S. aureus clinical isolates (19). The remaining two antigens, PmtA and PmtC, belong to Pmt ABC transporter family, an essential S. aureus toxin export system that delivers phenol-soluble modulins (PSMs) into host cells. Mutation of this system efficiently inhibits the growth and virulence of S. aureus (20). Immunization with either PmtA or PmtC protected mice from S. aureus lethal challenge, and both have shown great potential as effective vaccine candidates in various murine models (unpublished data and see Fig. S1 in the supplemental material). In summary, the five selected antigens target three key virulence and immunoevasion mechanisms employed by S. aureus in the infection process. Therefore, this multivalent vaccine was named Sta-V5 (for “5-valent S. aureus vaccine”).

10.1128/mSphere.00362-19.1 FIG S1 The protective efficacy of each component of Sta-V5 was assessed in the blood infection and peritonitis models. Mice vaccinated with each antigen were challenged with S. aureus USA300 and Newman strains, respectively. Survival data were analyzed by the log rank (Mantel-Cox) test. Download FIG S1, TIF file, 0.7 MB. Copyright © 2019 Deng et al. 2019 Deng et al. This content is distributed under the terms of the Creative Commons Attribution 4.0 International license.

### All components were conserved and expressed *in vivo*.

**FIG 1:**
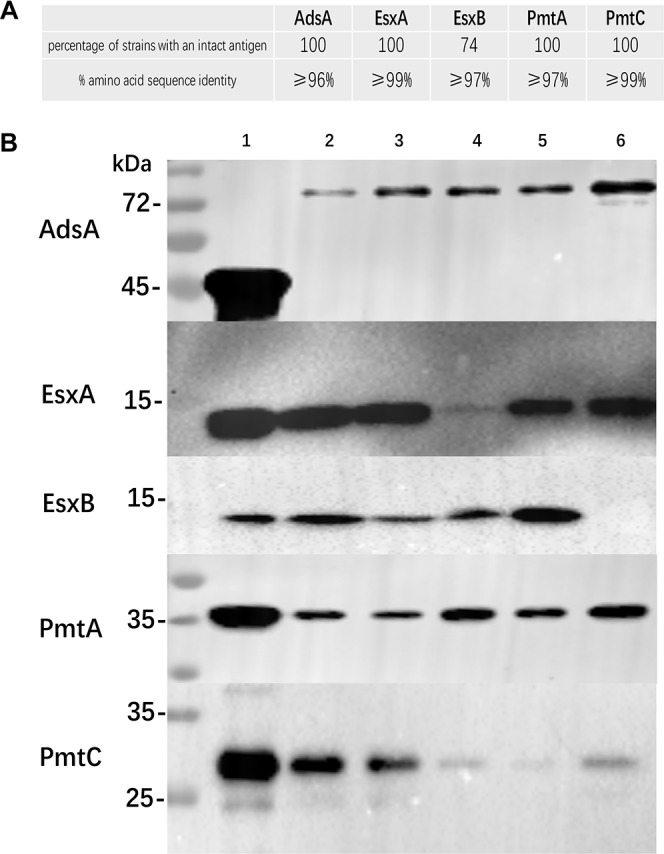
(A) Genetic alignment of *adsA*, *esxA*/*B*, and *pmtA*/*C* among 100 S. aureus strains. (B) *In vitro* expression of AdsA, EsxA/B, and PmtA/C was evaluated in five S. aureus isolates. Lane 1, His-tagged recombinant protein; lane 2, USA300; lane 3, Newman; lane 4, Staph1310; lane 5, Staph1510; lane 6, Staph1610. Expected molecular weight of antigens: rAdsA (AdsA28-422), 45.3 kDa (396 amino acids [aa]); AdsA, 83.5 kDa (772 aa); EsxA, 11 kDa (97 aa); EsxB, 11.5 kDa (104 aa); PmtA, 34.5 kDa (298 aa); PmtC, 33 kDa (290 aa).

Analyzing 100 S. aureus genome sequences available in NCBI databases, we found that genes *adsA*, *esxA*, *pmtA*, and *pmtC* were highly conserved in all strains selected. The level of protein sequence identity ranged from 96% to 100% (Fig. 1A). Gene *esxB* was missing in 26% of the isolates analyzed, but the level of amino acid sequence identity of *esxB* was high when present in the genome. To investigate the expression of the five antigens *in vivo*, we performed Western blot analysis on cell extracts of five epidemiologically relevant S. aureus strains. As shown in Fig. 1B, AdsA, PmtA and PmtC, and EsxA were expressed in all five strains. EsxB was not expressed in Staph1610 as the *esxB* gene is missing.

### Sta-V5 mounts consistent protective immunity against epidemiologically relevant staphylococcal strains in different mouse models.

**FIG 2:**
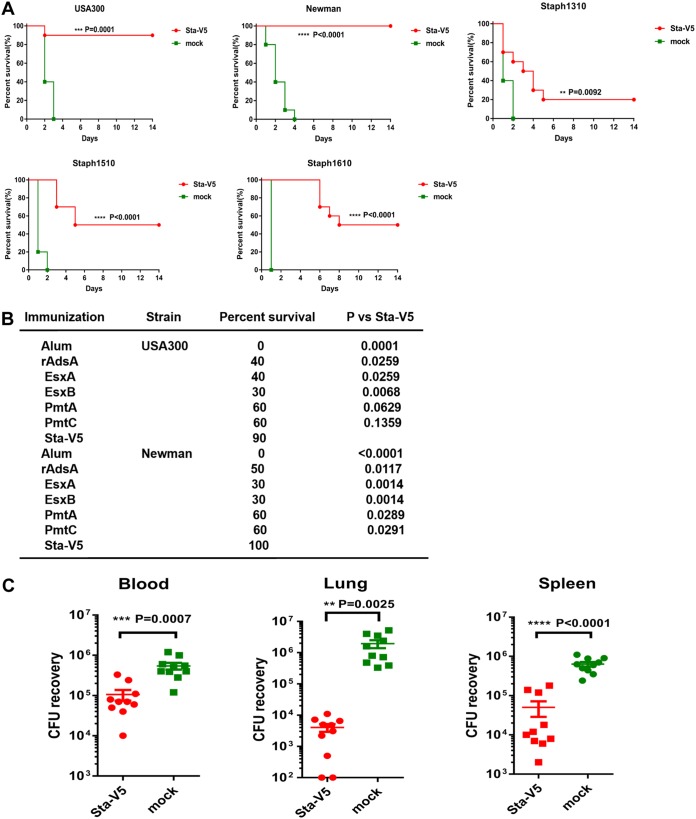
Sta-V5 induces protection against clinically relevant S. aureus strains in the blood infection model. (A) Mice were challenged with a lethal dose of USA300, Newman, Staph1310, Staph1510, or Staph1610 (*n* = 10, two independent experiments). (B) Comparison of efficacy of Sta-V5 with that of single antigens at increasing survival rates in the blood infection model. Survival curves representing single-antigen immunization results are presented in Fig. S1. (C) The bacterial load in different organs of mice after intravenous challenge with a sublethal dose of USA300. Statistical analysis was performed by log rank (Mantel-Cox) test (A) and by unpaired *t* test (B and C).

The antigens selected target three key virulence and immunoevasion mechanisms employed by S. aureus in the infection process, and each antigen was capable of inducing protection to various degrees in different mouse models of S. aureus infection (references 12, 13, and 19 and unpublished data). We hypothesized that the antigens could work in a synergistic manner and elicit broader and more effective protection when used in combination. To test this hypothesis, we immunized mice with each antigen alone or with Sta-V5. After immunization, mice were challenged intravenously with a lethal dose of five S. aureus strains, namely, strains USA300, Newman, Staph1310, Staph1510, and Staph1610. The survival of mice in a 14-day period was recorded. As shown in Fig. 2A, immunization with Sta-V5 resulted in a significantly increased survival rate compared with control mice immunized with only adjuvant (mock) regardless of the strains used. More than 90% of the Sta-V5-immunized mice survived over 14 days after lethal challenge with strain USA300 or strain Newman. The survival rate of mice immunized with a single antigen ranged from 30% to 60% but was significantly lower than that seen with mice immunized with Sta-V5 (Fig. S1). In addition, the bacterial load recovered from different organs in mice after intravenous challenge with a sublethal dose of strain USA300 was determined. As shown in Fig. 2B, immunization with Sta-5V resulted in a significant reduction of bacterial load compared with the level seen with control mice immunized with alum alone. The reduction of bacterial load was most significant in lungs.

**FIG 3:**
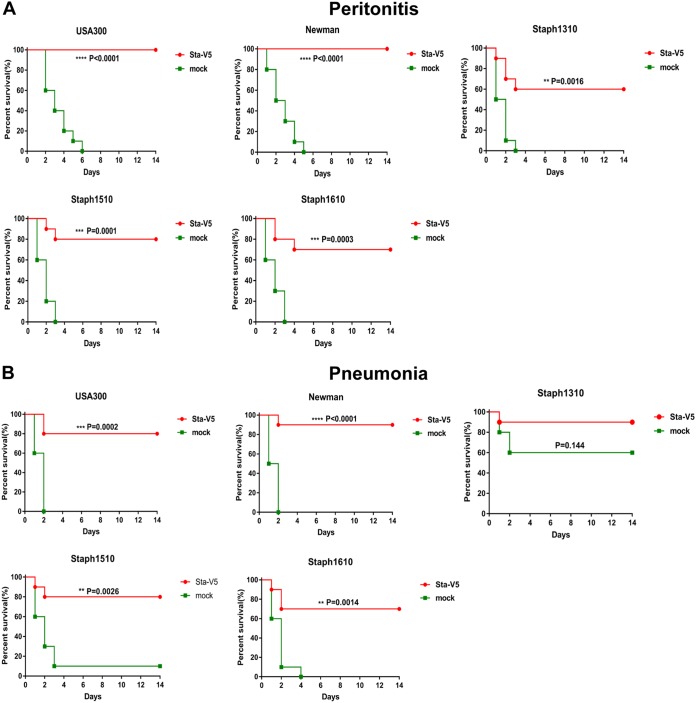
Sta-V5 elicits protective immunity against staphylococcal strains in peritonitis and pneumonia models. (A) In the peritonitis model, mice were challenged (i.p.) with a lethal dose of USA300, Newman, Staph1310, Staph1510, or Staph1610 (*n* = 10, two independent experiments). (B) In the pneumonia model, animals were subjected to nasal inoculation of USA300, Newman, Staph1310, Staph1510, or Staph1610 (*n* = 10, two independent experiments). Data were analyzed by log rank (Mantel-Cox) test.

We also evaluated the efficacy of Sta-V5 in the peritonitis infection model using the five strains mentioned above. As shown in Fig. 3A, a 100% survival rate was achieved over the 14-day period after mice immunized with Sta-V5 were subjected to challenge with either strain USA300 or strain Newman. Vaccination with Sta-V5 also significantly increased the number of mice surviving challenge with strain Staph1310, Staph1510, or Staph1610.

Next, we tested the protective efficacy of Sta-V5 in the pneumonia model. The survival of immunized and control mice was evaluated for a 14-day period after nasal challenge with strain USA300, Newman, Staph1310, Staph1510, or Staph1610. The survival rate of mice immunized with Sta-V5 was always significantly superior to that observed in mock-immunized mice for all strains, with the exception of the Staph1310 strain (Fig. 3B). The mortality in the control group reached only 40%, which contrasts with the fact that the members of the control group died in less than 3 days under conditions of challenge with other strains. This anomaly was probably due to the low level of expression of Staph1310 virulence factors with a predominant role in the pneumonia model.

**FIG 4:**
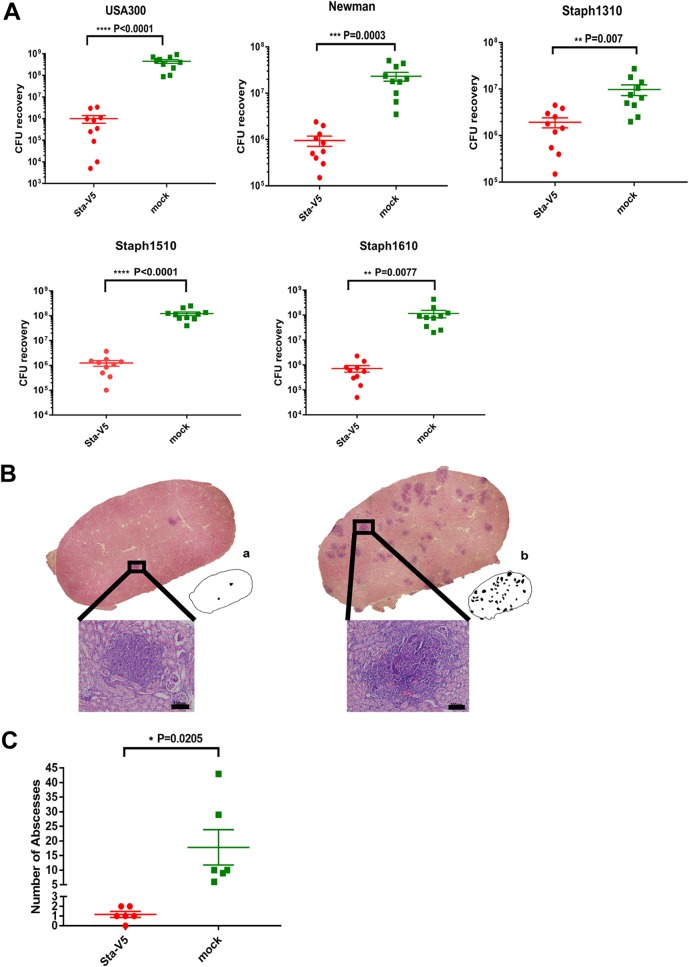
In the renal abscess model, S. aureus burden was significantly reduced in mice immunized with Sta-V5. (A) Bacterial load of kidneys removed from mice after challenge with USA300, Newman, Staph1310, Staph1510, or Staph1610 (*n* = 10, two independent experiments). (B) Representative images of kidney sections of S. aureus-infected mice immunized with Sta-V5 (left) and with Alum (right) by HE staining. Cartoon pictures (a and b) indicate the abscess area in the kidney sections described above. High magnification of the areas in the black boxes (insets) indicated that the staphylococcal abscess (center) was surrounded by infiltrating polymorphonuclear leukocytes. (C) Quantitative analysis of abscess formation in Sta-V5-immunized and mock-immunized mice infected with S. aureus USA300. The abscesses was enumerated in kidneys harvested from Sta-V5-immunized and mock-immunized mice.

We further evaluated the potential protective effect of Sta-V5 in a mouse renal abscess model. Four days after administration of a sublethal challenge dose of USA300, Newman, Staph1310, Staph1510, or Staph1610, mice were sacrificed, and the bacterial loads of their kidney homogenates were counted. The CFU level was significantly lower in kidneys collected from mice immunized with Sta-V5 for all five strains (Fig. 4A). Histological analysis of kidney from mice immunized with alum alone revealed a large number of abscesses with necrotic immune cells surrounding staphylococcal cells (Fig. 4B). In contrast, abscesses were barely found in kidneys collected from Sta-V5-immunized mice.

**FIG 5:**
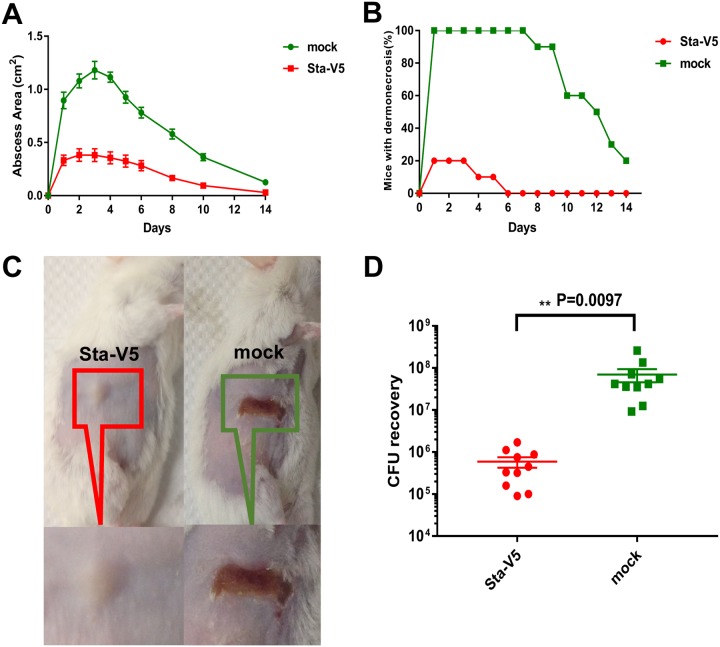
Sta-V5 immunization alleviates skin infections in mice after S. aureus inoculation. Active immunization with Sta-V5 reduced the size of abscesses (A) and the level of bacterial load (D) (*n* = 10, two independent experiments). (B) Vaccination of Sta-V5 also decreased the occurrence of dermonecrosis. (C) Representative images of mouse skin lesions (day 5). Results represent means ± standard errors of the means (SEM). Statistical analysis was performed by using an unpaired *t* test.

Finally, we used a skin infection model to assess the protective immunity induced by Sta-V5 against staphylococcal skin infection. Immunized and control mice were subjected to subcutaneous injection in the shaved right flank with S. aureus USA300 strain. Abscess mass and dermonecrotic area were monitored at 24-h intervals over a 14-day period. As a result, active immunization of Sta-V5 significantly reduced the abscess size. Moreover, dermonecrosis was dramatically absent in the vaccinated mice (Fig. 5).

### Sta-V5 induces protective antibodies and elicits robust skewed Th1/Th17 responses.

**FIG 6:**
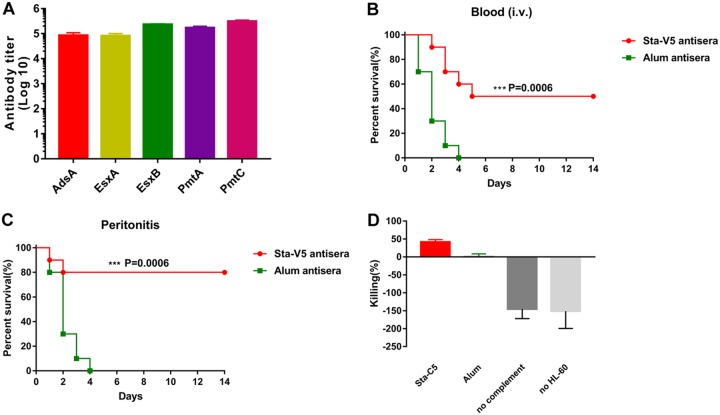
Sta-V5 induces effective antibodies. (A) High antibody titers against vaccine components were elicited in the tested mice. (B) Naive mice that received Sta-V5 antisera were more effectively protected against S. aureus USA300 challenge in the blood infection model. i.v., intravenous. (C) In the peritonitis model, the results obtained were similar to those determined as described for panel A. (D) Sera against Sta-V5 mediated opsonophagocytosis of S. aureus USA300. Percent killing was calculated as the ratio between the percentages of killing achieved with and without sera. Sera from alum-treated mice (Alum) failed to mediate the killing. Likewise, no bacterial killing was observed in the absence of functional complement or HL-60 cells. Error bars represent standard deviations (SD). Data were analyzed by log rank (Mantel-Cox) test.

Active immunization of Sta-V5 elicited a robust immune response in mice (Fig. 6A). We asked if the antibodies were functional and if they contributed to the protection induced by the vaccine. To address that issue, we conducted passive-protection experiments using rabbit anti-Sta-V5 serum. Before naive mice were intravenously challenged with a lethal dose of S. aureus USA300 strain, serum samples were administered to animals through intravenous injection. As shown in Fig. 6A, 50% of the mice that received anti-Sta-V5 serum survived for 14 days. In contrast, all control mice were killed in 4 days. Further, the survival rate of passively immunized mice subjected to intraperitoneal (i.p.) S. aureus challenge was as high as 80% (Fig. 6B). To dissect the mechanism by which anti-Sta-V5 antibodies protected mice from S. aureus infection, we investigated if the Sta-V5 rabbit antiserum could facilitate opsonophagocytosis of S. aureus USA300 strain. The antiserum samples were incubated with S. aureus USA300 and differentiated HL-60 cells in the presence of rabbit complement. As shown in Fig. 6D, serum from alum-immunized rabbit did not mediate bacterial killing. Likewise, bacterial killing was not facilitated without HL-60 cells or active rabbit complement. In contrast, HL-60 cells killed over 40% of S. aureus cells with the Sta-V5 antiserum.

**FIG 7:**
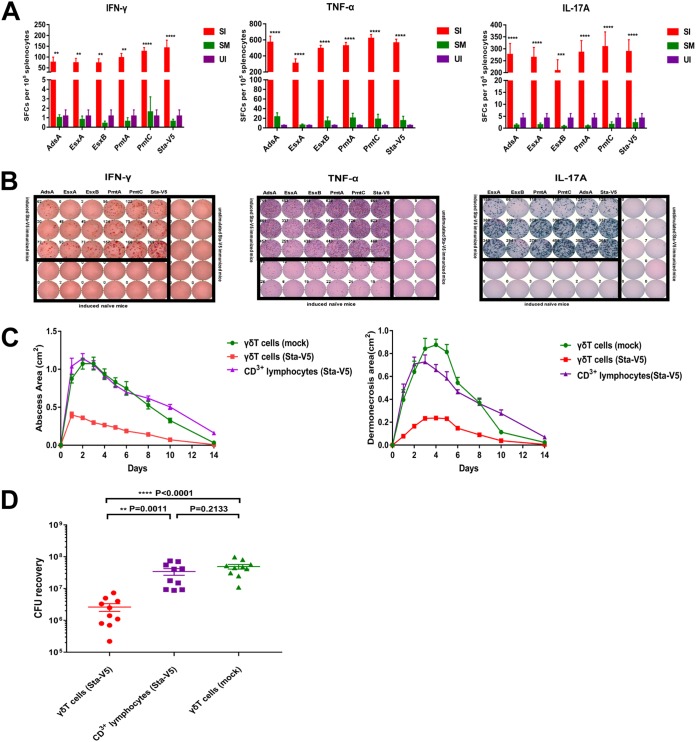
Robust IFN-γ, TNF-α, and IL-17A responses were elicited by Sta-V5 immunization. (A) Significant amounts of IFN-γ-, TNF-α-, and IL-17A-producing splenocytes from immunized mice were detected by ELISPOT assay. (B) Representative images of wells with IFN-γ, TNF-α, and IL-17A-producing splenocytes are shown. Data are presented as means ± SD. SFC, spot-forming cells; SI, immunized mice stimulated with antigens alone or in combination; SM, mock-immunized mice stimulated with antigens alone or in combination; UI, unstimulated immunized mice. (C and D) γδ T cells play an important role in protection conferred by Sta-V5 in the skin infection model. After S. aureus inoculation, the sizes of skin abscesses and dermonecrotic lesions (C) and total bacterial loads (D) were significantly reduced in naive mice administered 50,000 γδ T cells isolated from vaccinated mice. No protection was observed in mice that received 100-fold CD3^+^ T cells (devoid of γδ T cells). Data are presented as means ± SD.

We next analyzed antigen-specific interferon gamma (IFN-γ), tumor necrosis factor alpha (TNF-α), and interleukin-17A (IL-17A) T-cell responses by enzyme-linked immunosorbent spot (ELISPOT) assay. IFN-γ, TNF-α, and IL-17A play essential roles in the host protective immunity against S. aureus infection. Mice immunized with Sta-V5 were sacrificed after the third boost, and splenocytes were collected. As shown in Fig. 7A, the number of IFN-γ-producing splenocytes was much higher in vaccinated mice with the induction of antigens, alone or in combination. In parallel with this result, TNF-α-producing and IL-17A-producing splenocytes from mice immunized with Sta-V5 significantly outnumbered those from control mice in response to stimulation with the combined vaccine proteins or with each protein alone.

### γδ T cells from vaccinated mice conferred protection against S. aureus challenge in a skin infection model.

Results of previous studies have implied that γδ T cells play a pivotal role in long-lasting immune protection against S. aureus skin infection (21). In this study, we asked if γδ T cells contributed to the protection induced by Sta-V5. In particular, total lymphocytes were obtained from draining lymph nodes in Sta-V5-vaccinated mice. γδ T cells were isolated and administered to mice through intravenous injection 24 h before animals were subjected to subcutaneous challenge with the S. aureus USA300 strain. As shown in Fig. 7, administration of γδ T cells alleviated the S. aureus skin infection as both abscess and lesion size were significantly reduced compared with the results seen with control mice (mice that had received γδ T cells from naive mice). Interestingly, injection of 100-fold CD3^+^ lymphocytes devoid of γδ T cells isolated from vaccinated mice did not mediate protection against S. aureus skin infection.

## DISCUSSION

Being a frequent inhabitant of the human body, S. aureus causes serious invasive diseases and its infection represents a high unmet medical need. It is estimated that 5% of patients in U.S. hospitals carry MRSA in their nose or on their skin (data from CDC). With the growing threat of antibiotic resistance, the administration of vaccine would be an important additional preventative approach. However, developing an effective vaccine has proven to be difficult. S. aureus is not only very adept at evading host immune system but also dedicated to expressing a plethora of toxins that attack host immune cells. Therefore, our rationale for designing an effective vaccine against S. aureus infection was (i) to include conserved antigens to confer broad protection, (ii) to select antigens that play different roles in pathogenesis to stop the early infectious process by inducing high levels of both functional antibodies and protective T-cell responses, (iii) to employ different animal models to enhance the reliability and predictive value of the study, and (iv) to test a variety of clinical strains of S. aureus. As a result, to formulate our vaccine Sta-V5, we selected (i) AdsA, a recently discovered immune evasion factor; (ii) EsxA and EsxB, which modulate host cell apoptosis; and (iii) PmtA and PmtC, members of an essential S. aureus toxin export system that delivers phenol-soluble modulins (PSMs) into host cells. Initially, the formulation included only rAdsA and EsxA/B, as our group demonstrated previously that AdsA-specific antisera effectively facilitated opsonophagocytic killing of S. aureus and that EsxA/B induced robust Th17-based immune responses (13, 19). The addition of PmtA/C to the formulation significantly improved the protective efficacy in murine blood infection, peritonitis, and skin infection models (unpublished data). Although PmtA and PmtC are associated with an essential toxin pump that secrets PSMs, the underlying mechanism that accounts for how PmtA and PmtC work as effective vaccines is still under study in our laboratory. In this study, the five antigens conferred to vaccine Sta-V5 superior capabilities of protecting vaccinated animals from challenge by multiple genetically different S. aureus isolates. Notably, ∼90% of immunized mice survived over a 14-day period after challenge with an intravenous lethal dose of USA300 or Newman. Likewise, the survival rate of animals vaccinated with Sta-V5 reached as high as 100% in the peritonitis model. None of the single antigens conferred comparable protective immunity, implying the anticipated synergy between the individual antigens (see Fig. S1, S3, S4, and S5 in the supplemental material). Plus, the combined vaccine Sta-V5 elicited effective protection in all five different murine disease models. In addition to murine models, Sta-V5 also induced a robust antibody response and protective immunity in dairy cattle (unpublished data).

10.1128/mSphere.00362-19.2 FIG S2 The efficacy of Th1-mediated and Th17-mediated protection was investigated in the blood infection model. Mice immunized with Sta-V5 were intravenously injected with 100 mg of anti-IL-17A antibody 1 day before and 1 day and 4 days after USA300 challenge. Survival data were analyzed by log rank (Mantel-Cox) test. Download FIG S2, TIF file, 0.4 MB. Copyright © 2019 Deng et al. 2019 Deng et al. This content is distributed under the terms of the Creative Commons Attribution 4.0 International license.

10.1128/mSphere.00362-19.3 FIG S3 Protective efficacy of rAdsA, EsxA, and EsxB in pneumonia models. Animals were subjected to nasal inoculation of USA300 (*n* = 10, two independent experiments). Data were analyzed by log rank (Mantel-Cox) test. Download FIG S3, TIF file, 0.4 MB. Copyright © 2019 Deng et al. 2019 Deng et al. This content is distributed under the terms of the Creative Commons Attribution 4.0 International license.

10.1128/mSphere.00362-19.4 FIG S4 CFU recovery of kidneys in mice immunized with rAdsA, EsxA, and EsxB. Animals were subjected to sublethal doses of USA300 (*n* = 10, two independent experiments). Data were analyzed by unpaired *t* test. Download FIG S4, TIF file, 0.3 MB. Copyright © 2019 Deng et al. 2019 Deng et al. This content is distributed under the terms of the Creative Commons Attribution 4.0 International license.

Previous studies have emphasized that three mechanisms play important roles in eliminating Staphylococcus aureus: (i) Th1 responses that induce a high level of IFN-γ (22), (ii) Th17 responses that create a proinflammatory environment via the activity of IL-17A (23), and (iii) high titers of functional antibodies that mediate phagocytosis of S. aureus and blocking of virulence effectors (12, 19). In the current study, robust levels of secretion of IFN-γ and IL-17A were detected in splenocytes isolated from vaccinated mice when they were stimulated by each of the antigens alone or by their combination. The results indicated that Sta-V5 mounted a well-established Th1/Th17 skewed response which facilitated the clearance of S. aureus. Note that elimination of IL-17A or IFN-γ by intravenous injection of anti-IL-17A or anti-IFN-γ antibody did not have a significant impact on the survival of Sta-V5-vaccinated mice subjected to lethal challenge by S. aureus in a blood infection model (Fig. S2). However, the importance of IL-17A and IFN-γ could not be denied on the basis of this result as levels of IL-17A or IFN-γ might not be completely depleted in mice due to insufficient titers of antibodies administered in the blood infection model. On the other hand, Sta-V5 also induced functional antibodies that not only increased the rate of survival of naive mice upon S. aureus challenge but also promoted opsonophagocytosis in the presence of HL-60 cells and rabbit complement. In addition, there is increasing evidence implying that “alternative” T cells could provide a novel strategy for next-generation antistaphylococcus vaccine design (24). In the current study, we demonstrated that the protection induced by Sta-V5 was at least partly mediated by γδ T cells in the skin infection model. In contrast, intravenous injection of a 100-fold volume of CD3^+^ lymphocytes from vaccinated mice failed to produce any protective effect. This is in line with the recent finding that γδ T cells represent a mechanism for long-lasting immunity against recurrent S. aureus skin infections (21). Previous studies demonstrated that IL-17-producing and/or IFN-γ-producing memory T cells may be essential to vaccine protection at sites of infection (25, 26). Therefore, the roles of IFN-γ, IL-17, and other cytokines in adaptive immunity induced by Sta-V5 through γδ T cells warrant further study.

Finally, we analyzed sera of patients infected by MRSA for their reactivity against the purified vaccine proteins to investigate if the antigens selected were expressed and immunogenic during infection. It was found that antibodies against the antigens were detected in the majority of 12 patients who suffered from S. aureus bacteremia. Some serum samples even recognized all five antigens in Western blot analysis (Fig. S6A and B).

10.1128/mSphere.00362-19.5 FIG S5 CFU recovery of skin abscesses in mice immunized with rAdsA, EsxA, and EsxB. Animals were subjected to challenge with USA300 (*n* = 10, two independent experiments). Data were analyzed by unpaired *t* test. Download FIG S5, TIF file, 0.3 MB. Copyright © 2019 Deng et al. 2019 Deng et al. This content is distributed under the terms of the Creative Commons Attribution 4.0 International license.

10.1128/mSphere.00362-19.6 FIG S6 (A) Antibodies against Sta-V5 components were detected in 12 serum samples of patients suffering from S. aureus bacteremia. Those patients recovered from infection eventually. (B) Representative Western blot of serum collected from patients. Download FIG S6, TIF file, 1.4 MB. Copyright © 2019 Deng et al. 2019 Deng et al. This content is distributed under the terms of the Creative Commons Attribution 4.0 International license.

## MATERIALS AND METHODS

### Bacterial strains, media, and growth conditions.

The bacterial strains used in this study are listed in Table S1 in the supplemental material. S. aureus clinical isolates Staph1310, Staph1510, and Staph1610 were provided by R. Y.-T. Kao. S. aureus strains were grown at 37°C in brain heart infusion (BHI) broth. Before bacterial challenge, overnight cultures of S. aureus strains were subcultured and harvested until an optical density at 600 nm (OD_600_) of approximately 1 was reached. The bacteria were then collected by centrifugation, washed once, and resuspended in phosphate-buffered saline (PBS).

10.1128/mSphere.00362-19.7 TABLE S1 Bacterial strains. Download Table S1, PDF file, 0.05 MB. Copyright © 2019 Deng et al. 2019 Deng et al. This content is distributed under the terms of the Creative Commons Attribution 4.0 International license.

For expression of recombinant proteins, Escherichia coli was grown in Luria Bertani broth containing 100 μg/ml ampicillin or 40 μg/ml kanamycin to an OD_600_ of ∼0.5. A 1-liter volume of bacterial culture was induced with 0.5 to 1 mM isopropyl-β-d-1-thiogalactopyranoside (IPTG) and grown for 4 h at 37°C.

### Vaccine antigen gene analysis.

The genome sequences of 100 S. aureus isolates were downloaded from the National Center for Biotechnology Information database. Sequence alignment analyses were conducted by the use of ClustalW.

### Multilocus sequencing typing.

The seven housekeeping genes—*arcc*, *aroe*, *glpf*, *gmk*, *pta*, *tpi*, and *yqil*—of S. aureus were amplified by PCR using the primers provided at saureus.mlst.net (see Table S2). The sequencing results were uploaded to saureus.mlst.net for sequence type (ST) determination.

10.1128/mSphere.00362-19.8 TABLE S2 Primers. Download Table S2, PDF file, 0.04 MB. Copyright © 2019 Deng et al. 2019 Deng et al. This content is distributed under the terms of the Creative Commons Attribution 4.0 International license.

### Expression analysis of antigens.

The expression of vaccine antigens was investigated by Western blotting.

Overnight cultures of S. aureus strains were subcultured and harvested by centrifugation until an OD_600_ of ∼1 was reached. Bacteria were washed once with PBS and resuspended in 20 mM Tris buffer (pH 7.5) with protease inhibitor cocktails and 200 μg/ml lysostaphin. After incubation for 1 h at 37°C, the samples were subjected to five cycles of freeze and thaw. The resulting lysates were then separated by SDS-PAGE and transferred to nitrocellulose membranes. The membranes were probed with rabbit antisera, and the proteins were detected by chemiluminescence using TMB (3,3′,5,5′-tetramethylbenzidine) substrate. Rabbit antisera against single antigens were used.

The serum samples of patients who had recovered from MRSA bacteremia were collected 90 days after MRSA diagnosis in Queen Mary Hospital, Hong Kong. The presence of specific antibodies against vaccine antigens in human sera collected from patients with MRSA bacteremia was evaluated by immunoblot analysis using purified recombinant proteins. Five recombinant proteins purified from E. coli (100 ng each) were subjected to SDS-PAGE and transferred to nitrocellulose membranes. The membranes were then incubated with human sera at a 1:1,000 dilution. The immunoreactive signals were detected by chemiluminescence as previously described.

### Cloning and purification of antigens.

DNA sequences of the five antigens were amplified by PCR using genomic DNA of S. aureus strain USA300. The PCR products were then cloned into pET21a or pET28a vector. Next, the recombinant plasmids were transformed into E. coli BL21(DE3). Bacterial pellets harvested from 1 liter of bacterial culture were harvested by centrifugation and resuspended in binding buffer (Tris-HCl [pH 8.0], 20 mM imidazole) followed by sonication. After centrifugation at 15,000 × *g* for 30 min at 4°C, the soluble cell extracts were collected and filtered using a 0.22-μm-pore-size membrane. The samples were then loaded on a nickel-activated chelating Sepharose column (GE Healthcare). After washing, the bound proteins were eluted with elution buffer (Tris-HCl [pH 8.0], 250 mM imidazole). The purified proteins were identified using SDS-PAGE analysis and subjected to endotoxin removal afterward (Pierce).

### Active immunization.

Sta-V5 vaccine formulated with aluminum hydroxide gel adjuvant (Alum; 1 mg/ml) was injected intramuscularly (i.m.) or intraperitoneally (i.p.) into BALB/c mice (10 per group). The mice were subjected to booster vaccinations every 2 weeks (administered twice). A Sta-V5 formulation of 100 μl (i.m.) or 200 μl (i.p.) with 15 μg of each purified protein absorbed to Alum was used to immunize animals. Mice of the mock-immunized group received an equal amount of Alum in a mixture with PBS. The antibody titers to each antigen in the serum samples collected from animals were documented by enzyme-linked immunosorbent assay (ELISA) after each booster vaccination as described elsewhere.

### Passive immunization.

Rabbit immune sera (150 μl) were injected into the tail vein of 8-week-old BALB/c mice 24 h prior to challenge with the S. aureus strains. Equal volumes of sera collected from rabbits immunized with Alum were administered to control mice.

### Blood infection model.

Actively immunized (10 days after second booster vaccination, i.m.) or passively immunized BALB/c mice were challenged with 100 μl of a lethal or sublethal dose of S. aureus strains by tail vein intravenous injection. For the lethal dose challenge, mice were infected with 5 × 10^7^ CFU of USA300, Newman, Staph1510, or Staph1610. Staph1310 was administered in 3.5 × 10^7^ CFU. The well-being of infected mice was monitored daily for 14 days. The mice were sacrificed when a humane endpoint was reached or when they had suffered a >20% weight loss. For sublethal dose challenge, animals were injected with 2 × 10^7^ CFU of USA300. The blood samples were collected within 6 h after the sublethal challenge. The spleens and lungs were removed 4 days after the sublethal challenge and homogenized. CFU were enumerated following serial diluting and plating on BHI agar. In cytokine depletion experiments, anti-mouse IFN antibody (clone R4-6A2), anti-mouse IL-17A antibody (clone 17F3), and the corresponding isotype control antibodies were purchased from Bio X Cell. Mice received 300 μg of antibodies or isotype control via intravenous injection 1 day prior to infection and on days 1 and 4 postinoculation.

### Peritonitis infection model.

Actively immunized (10 days after second booster immunization, i.p.) or passively immunized BALB/c mice were challenged by 200 μl intraperitoneal injection of S. aureus strains. Animals were infected with 2 × 10^8^ to 5 × 10^8^ CFU of S. aureus strains (the inoculum was adjusted depending on the strains) and monitored daily for 14 days. The mice were sacrificed when a humane endpoint was reached or when the mice had suffered a >20% weight loss.

### Murine pneumonia model.

S. aureus strains USA300, Newman, Staph1310, Staph1510, and Staph1610 were used in the pneumonia model. After anesthetization was performed with ketamine (100 mg/kg of body weight) and xylazine (5 mg/kg), actively immunized (i.m.) or naive BALB/c mice were challenged intranasally with 3 × 10^8^ CFU per 30 μl of S. aureus strains. The well-being of infected mice was monitored daily for 14 days. The mice were sacrificed when a humane endpoint was reached or when the mice had suffered a >20% weight loss.

### Renal abscess model.

The preparation of bacteria for sublethal challenge was performed as described above. S. aureus strains (1 × 10^7^ to 2 × 10^7^ CFU) were intravenously injected into BALB/c mice on day 10 after the second booster vaccination (i.m.). Four days after bacterial challenge, infected mice were euthanized, and kidneys were collected and homogenized in 0.01% Triton X-100. The homogenates were diluted and plated on BHI agar for CFU enumeration. For histological analysis, kidney tissue samples were fixed in 10% formalin for 24 h at room temperature. Paraffin-embedded tissue blocks were sectioned at 5 μm and stained with hematoxylin-eosin to facilitate visualization. Stained sections were examined under a microscope.

### Murine skin infection model.

Experiments were performed as previously described. Female immunized (i.m.) or naive BALB/c mice were anesthetized and inoculated by subcutaneous injection in the right shaved ﬂank with 1 × 10^7^ CFU of S. aureus USA300 in 50 μl. The mass and abscess formation were monitored daily over 14 days. The size of abscesses and of associated overlying dermonecrotic lesions was determined by a standard equation as follows: *A* (area) = (π/2) × length × width. For CFU counting of S. aureus in skin abscess lesions, animals were euthanized 4 days after bacterial inoculation. Abscesses were removed and homogenized in 0.01% Triton X-100. CFU counting was performed by plating serially diluted samples on BHI agar at 37°C for overnight growth.

### Opsonophagocytosis killing assay.

The opsonophagocytosis killing assay (OPKA) was performed as previously described (27). Human promyelocytic leukemia HL-60 cells were differentiated into phagocytes using 0.8% dimethylformamide (DMF). S. aureus USA300 cells grown overnight were washed once in PBS and resuspended in Hanks’ balanced salt solution (HBSS) buffer (Ca^2+^/Mg^2+^). The bacteria were incubated with heat activated mouse antiserum against Sta-V5 at 4°C for 20 min. Differentiated HL-60 cells were distributed at 3.7 × 10^6^/well at a multiplicity of infection (MOI) of ∼50:1 in the presence of 10% (vol/vol) rabbit complement. Following incubation at 37°C for 1 h with agitation at 600 rpm, samples were plated on BHI agar plates for CFU enumeration.

### ELISPOT assay.

Animals were sacrificed 5 days after the second booster vaccination (i.m.). After euthanasia, spleens were collected and single suspensions of splenocytes were obtained. Interferon gamma (IFN-γ)-producing, tumor necrosis factor alpha (TNF-α)-producing, or interleukin 17A (IL-17A)-producing splenocytes from vaccinated or control mice were analyzed using a cytokine-specific enzyme-linked immunospot (ELISPOT) assay (R&D Systems, USA) as described by the manufacturer. Briefly, splenocytes isolated from immunized mice were plated at a concentration of 1 × 10^5^ cells/well and induced with each antigen alone (0.2 μg/well) or with the antigens in combination (0.1 μg of each antigen/well) in triplicate and incubated for 20 h at 37°C. Ionomycin (Sigma, USA) (1 μg/ml) and phorbol myristate acetate (PMA; Sigma) (50 ng/ml) were used as positive controls. Splenocytes from unstimulated, immunized mice and RPMI 1640-treated splenocytes were used as negative controls. After the cells were decanted, biotinylated primary monoclonal antibodies were added to each well and the plates were incubated for 1 h at 37°C. The plates were incubated with streptavidin-horseradish peroxidase (HRP) conjugate for 1 h at 37°C and subjected to color development with TMB solution. Finally, the spots were enumerated using an Immunospot analyzer.

### Transfer of γδ T cells to mice.

Animals immunized with Sta-V5 and Alum were euthanized 10 days after the booster vaccination. The draining lymph nodes were harvested and homogenized. A single suspension of lymphocytes was obtained by manually pushing draining lymph nodes through a cell strainer (40-μm pore size). Cells were enumerated and resuspended in PBS containing 0.5% bovine serum albumin (BSA) and 2 mM EDTA. γδ T cells were isolated using a TCRγ/δ+ isolation kit for mice (Miltenyi Biotec) according to the manufacturer’s instructions.

A total of 50,000 γδ T cells or 5,000,000 CD3^+^ T cells (devoid of γδ T cells) in the flowthrough were transferred to mice through intravenous injection at 24 h prior to S. aureus skin inoculation.

### Statistical analysis.

At least two independent experiments, run under the same conditions, were performed for all studies. For the blood infection, peritonitis, and pneumonia models, statistical significance was assessed with log rank (Mantel-Cox) analysis. The Student paired *t* test was used to analyze the statistical significance of results of OPKA experiments and of bacterial load measurements. T-cell responses to vaccination were analyzed using analysis of variance (ANOVA). Analyses were performed using GraphPad Prism 7 (GraphPad Software, USA), and a *P* value of <0.05 was considered statistically significant.

### Research ethics.

BALB/c mice and New Zealand rabbits were supplied by the Laboratory Animal Unit of the University of Hong Kong. All animal experiments were approved by the Committee on the Use of Live Animal in Teaching and Research of the University of Hong Kong (CULATR 4493-17).
