# Supplementary figures and images for: Broad and Effective Protection against Staphylococcus aureus Is Elicited by a Multivalent Vaccine Formulated with Novel Antigens
Source: mSphere. 2019 Sep 4;4(5):e00362-19. doi: 10.1128/mSphere.00362-19 (PMC6731528; doi:10.1128/mSphere.00362-19)

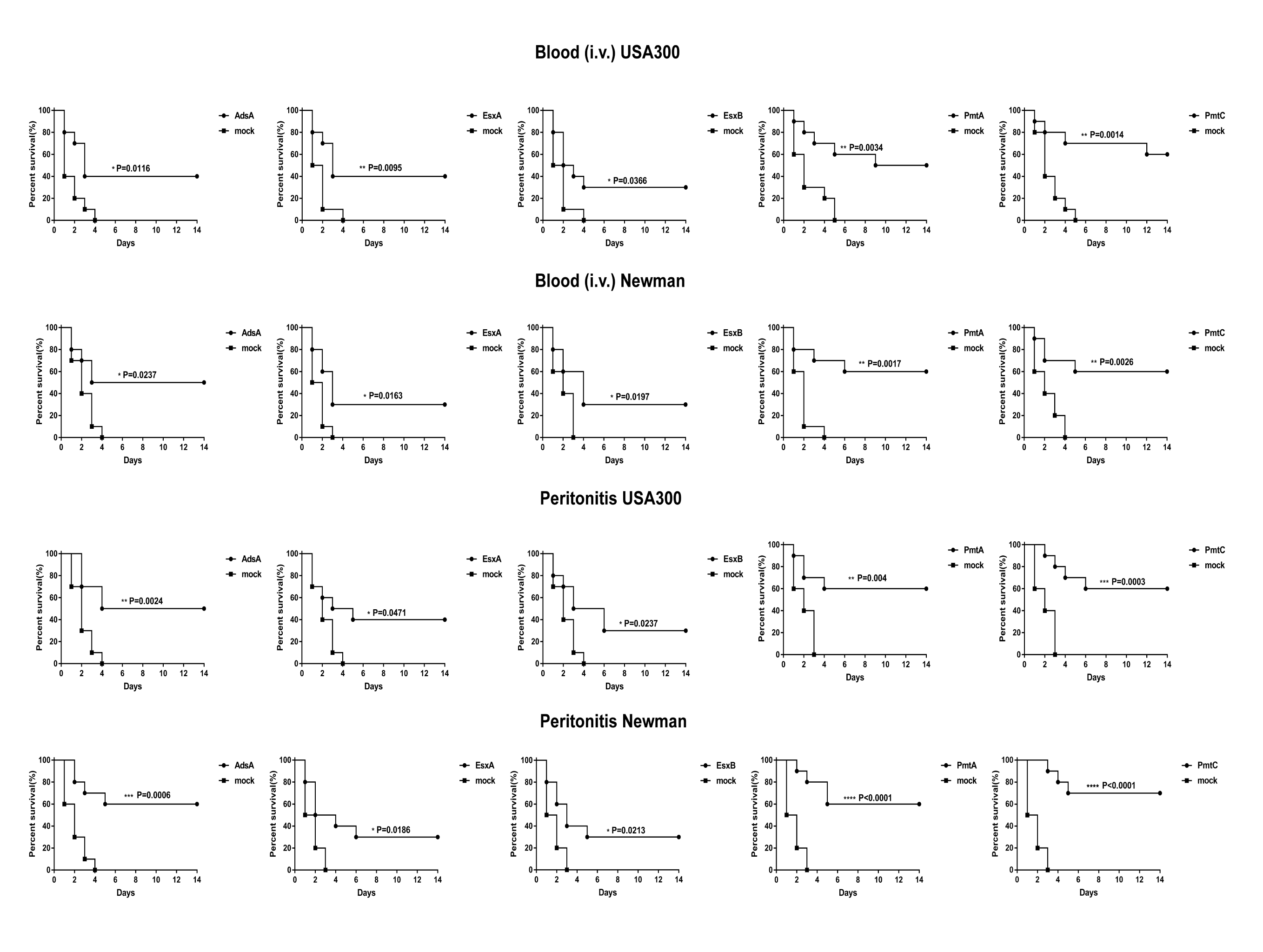

Supplement: FIG S1 [file mSphere.00362-19-sf001.tif]

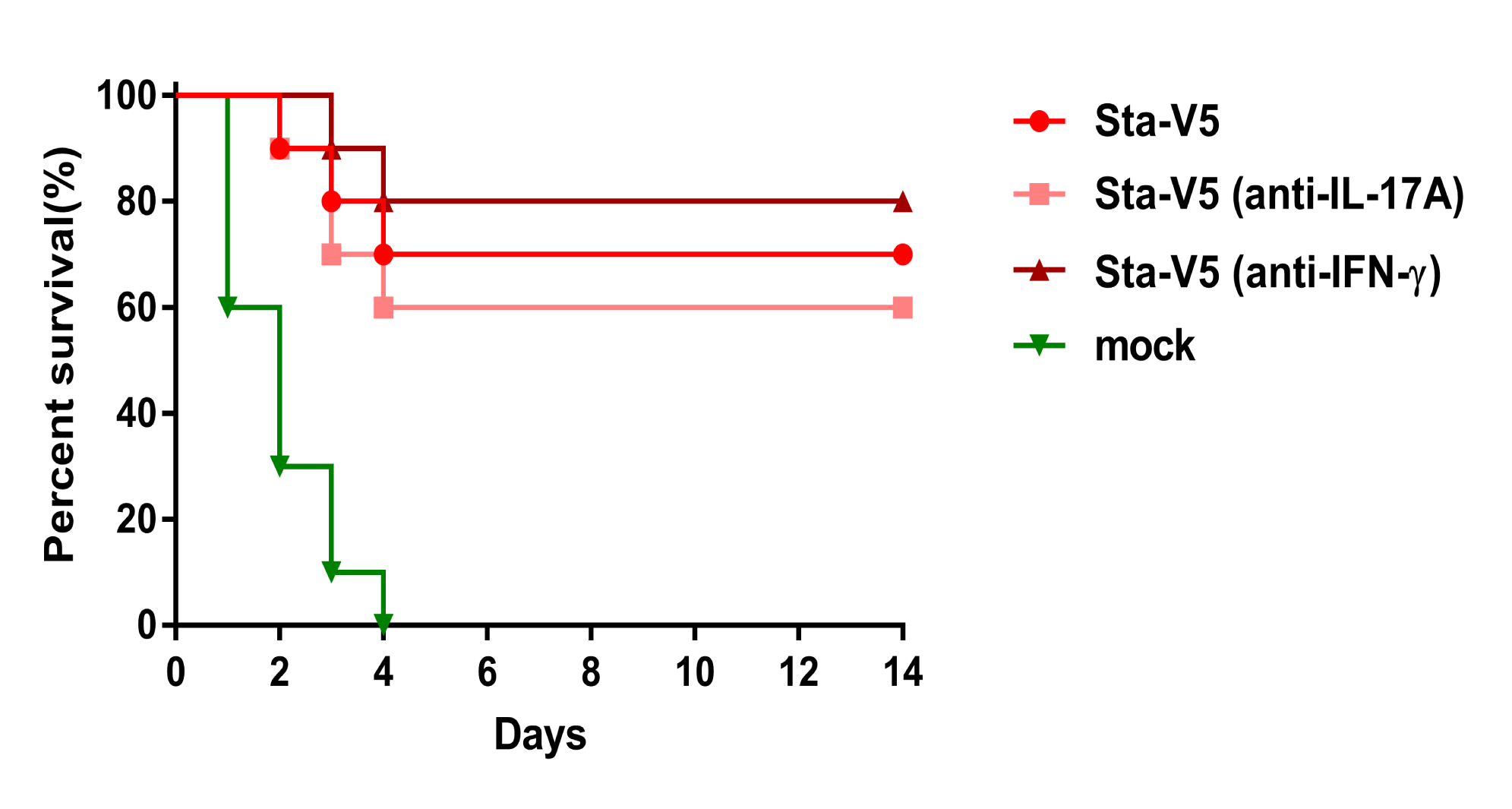

Supplement: FIG S2 [file mSphere.00362-19-sf002.tif]

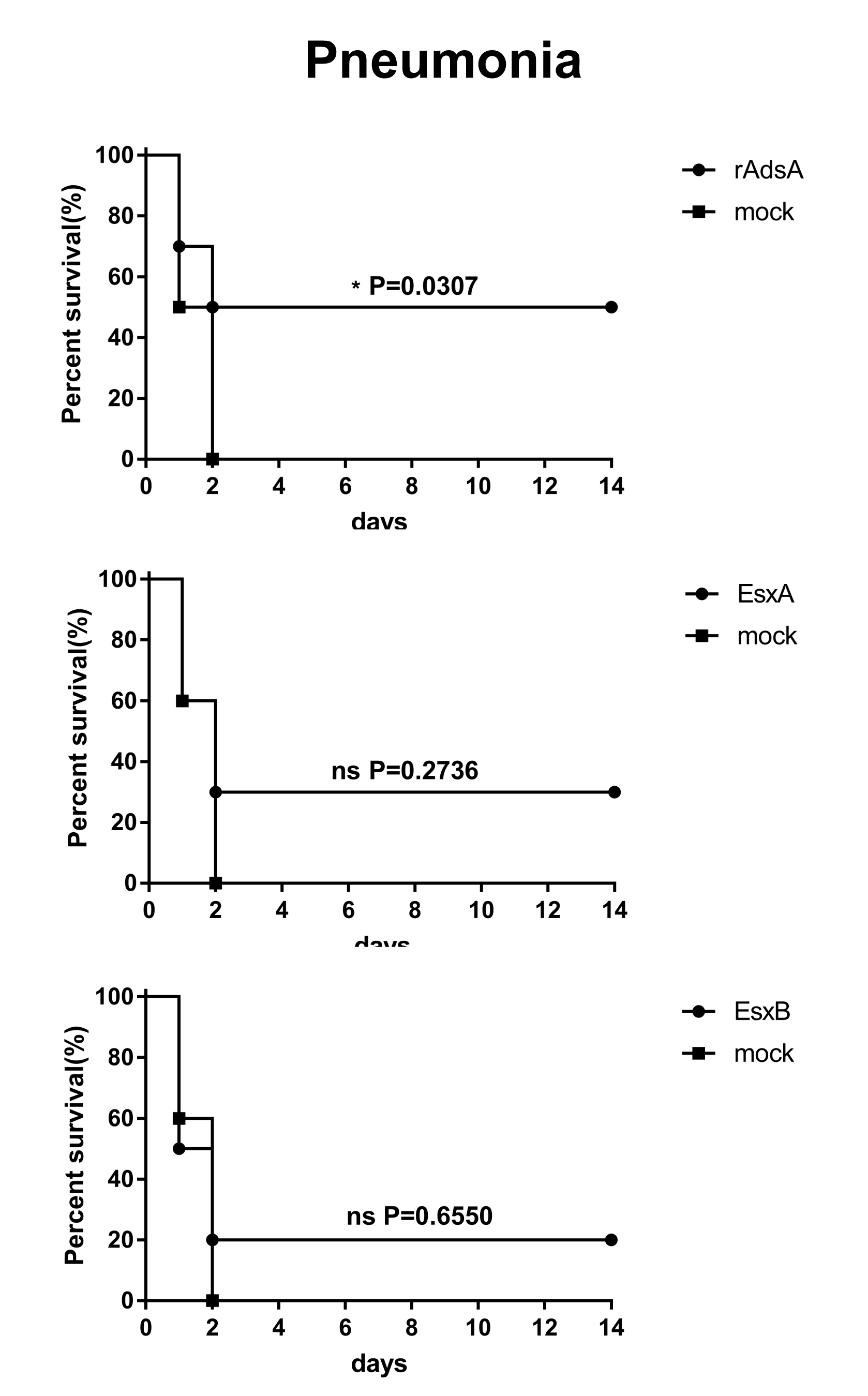

Supplement: FIG S3 [file mSphere.00362-19-sf003.tif]

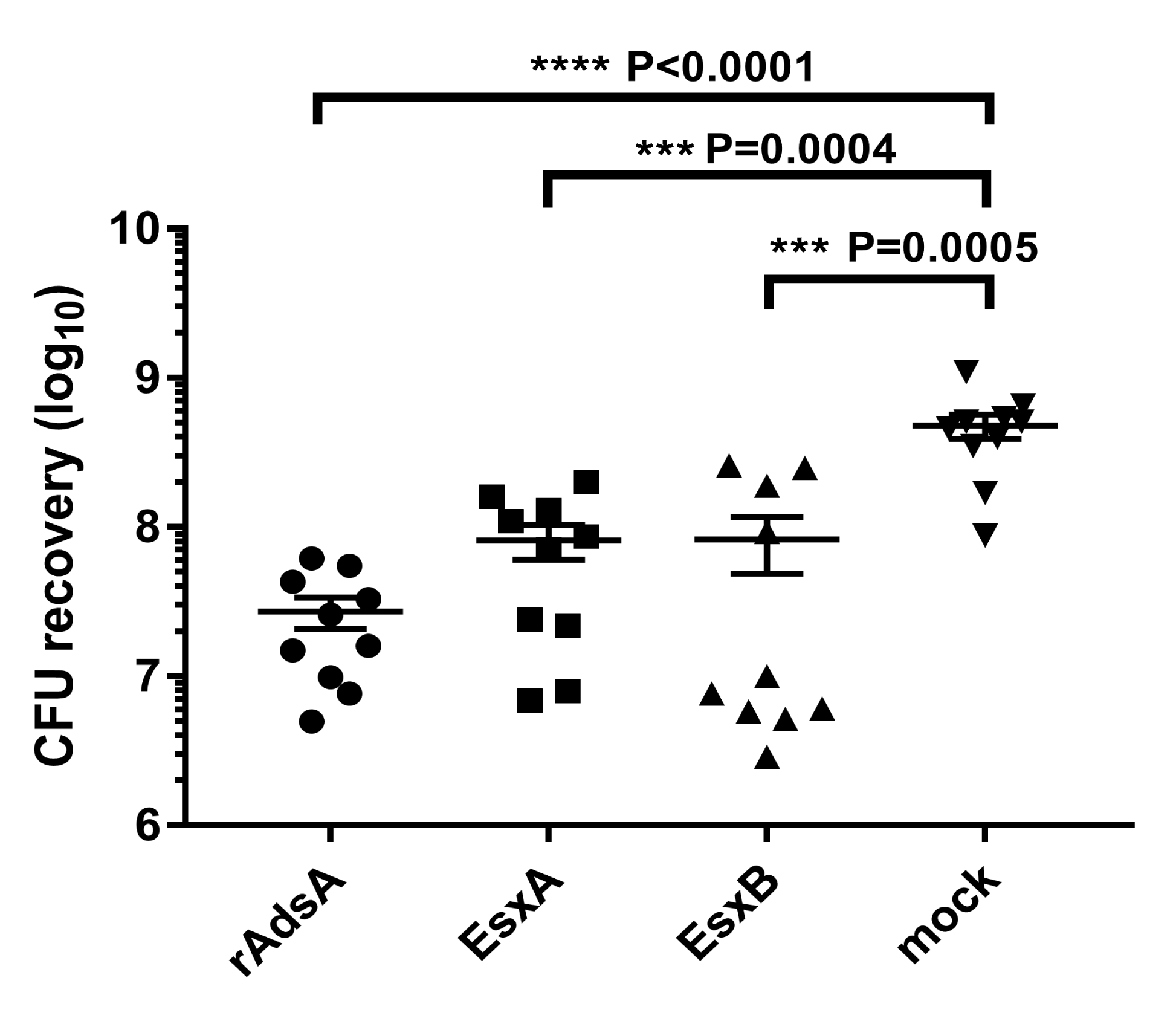

Supplement: FIG S4 [file mSphere.00362-19-sf004.tif]

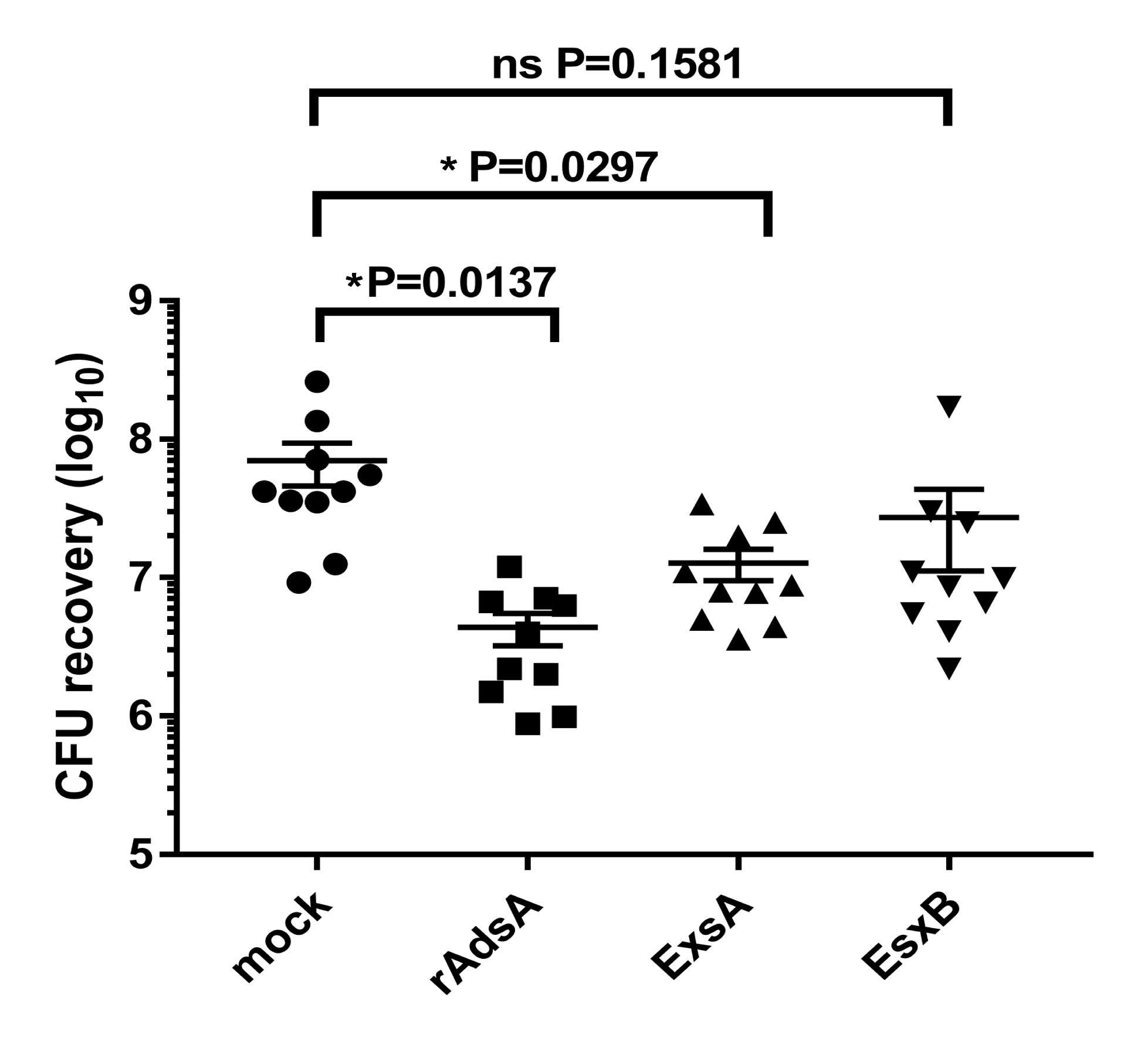

Supplement: FIG S5 [file mSphere.00362-19-sf005.tif]

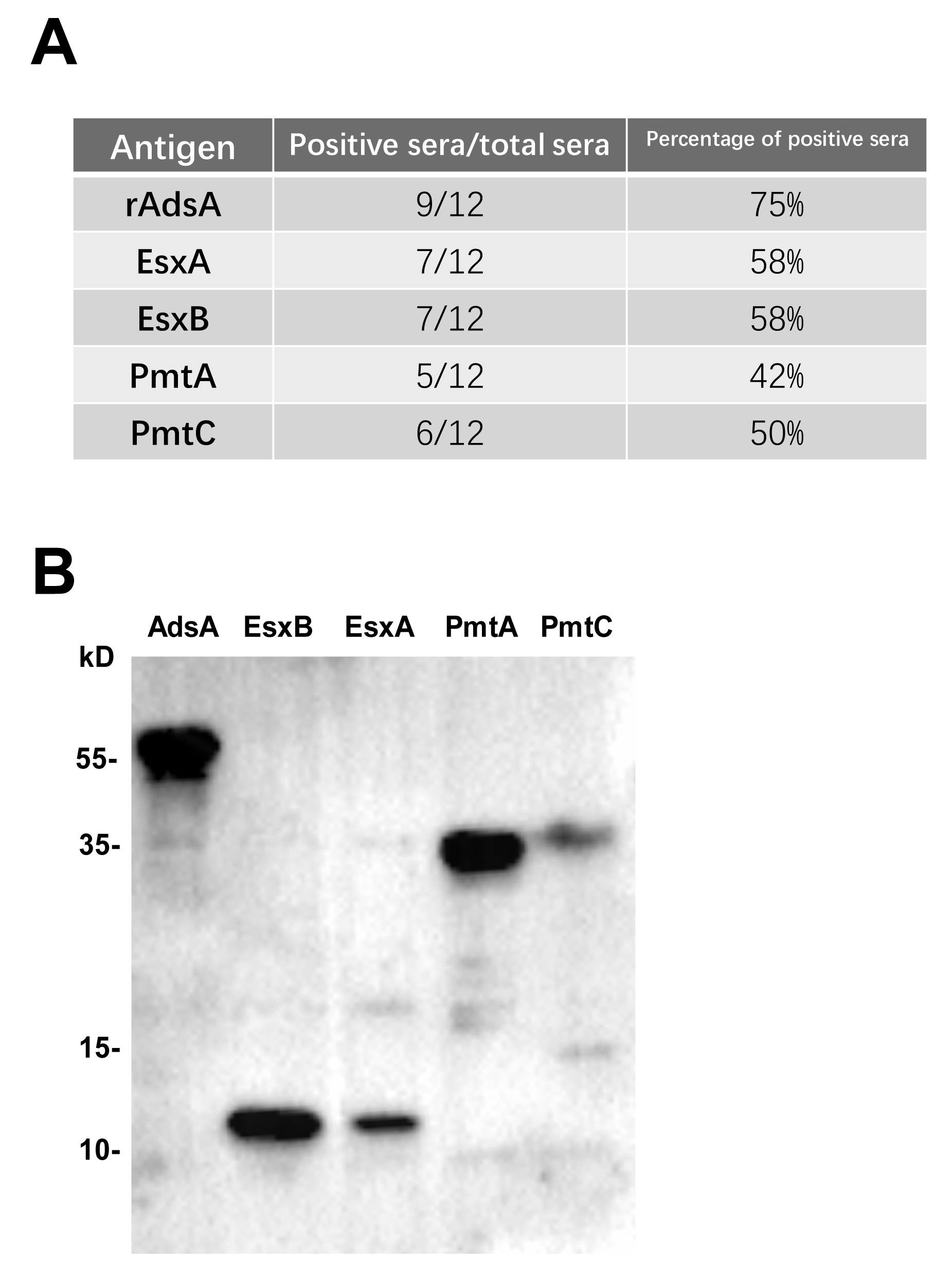

Supplement: FIG S6 [file mSphere.00362-19-sf006.tif]
