# Supplementary material for: Broad and Effective Protection against Staphylococcus aureus Is Elicited by a Multivalent Vaccine Formulated with Novel Antigens
Source: mSphere. 2019 Sep 4;4(5):e00362-19. doi: 10.1128/mSphere.00362-19 (PMC6731528; doi:10.1128/mSphere.00362-19)
Supplement: TABLE S1 [file mSphere.00362-19-st001.pdf]

| Strain | Characteristics | Source |
|---|---|---|
| BL21(DE) | Host strain for expression of antigens | Invitrogen |
| Newman | Clinical isolate, MSSA, ST254 | 1 |
| USA300 | Clinical isolate, MRSA, ST8 | This study |
| Staph 1310 | Clinical isolate, MRSA, ST1744 | This study |
| Staph 1510 | Clinical isolate, MRSA, ST398 | This study |
| Staph 1610 | Clinical isolate, MRSA, ST217 | This study |

MRSA, methicillin-resistant *S. aureus;* MSSA, methicillin-sensitive *S. aureus;* ST, sequence type

1. Baba T, Bae T, Schneewind O, Takeuchi F, Hiramatsu K (2008) Genome sequence of *Staphylococcus aureus* strain Newman and comparative analysis of staphylococcal genomes: polymorphism and evolution of two major pathogenicity islands. J Bacteriol 190(1):300–310.
