# Supplementary material for: Broad and Effective Protection against Staphylococcus aureus Is Elicited by a Multivalent Vaccine Formulated with Novel Antigens
Source: mSphere. 2019 Sep 4;4(5):e00362-19. doi: 10.1128/mSphere.00362-19 (PMC6731528; doi:10.1128/mSphere.00362-19)
Supplement: TABLE S2 [file mSphere.00362-19-st002.pdf]

**Primer**	**Sequence**	**Application**
rAdsA-F	aaacatatggctgagcaacatacaccaatgaa	PCR amplification of *radsA*
rAdsA-R	tacctcgagttgattaatttgttcagctaatgctttgt	PCR amplification of *radsA*
EsxA-F	aaacatatgatggcaatgattaagatgagt	PCR amplification of *esxA*
EsxA-R	tacctcgagaactttctaataatttcggtttgcaa	PCR amplification of *esxA*
EsxB-F	aaacatatgatgggtggatataaaggtattaaa	PCR amplification of *esxB*
EsxB-R	tacctcgaggcttgatagggtgaaccca	PCR amplification of *esxB*
PmtA-F	aaacatatgaatgccatagaattaagtaatgttaattat	PCR amplification of *pmtA*
PmtA-R	tacctcgagaaaaccttcttccatcaatttgatgttttc	PCR amplification of *pmtA*
PmtC-F	aaacatatggagaaaagagaggctaaattagaacat	PCR amplification of *pmtC*
PmtC-R	tacctcgagtttatcacctctttgatttatattgaagta	PCR amplification of *pmtC*
